# Supplementary material for: Upconversion-Powered Photoelectrochemical Bioanalysis for DNA Sensing
Source: Sensors (Basel). 2024 Jan 24;24(3):773. doi: 10.3390/s24030773 (PMC10856881; doi:10.3390/s24030773)
Supplement: Supplementary file 1 [file sensors-24-00773-s001.zip › sensors-2717411-supplementary.pdf]

Supplementary Materials

Upconversion-Powered Photoelectrochemical Bioanalysis for DNA Sensing

Hong Liu, Weiwei Wei, Jiajun Song, Jin Hu, Zhezhe Wang, Peng Lin

1. Materials

DNA sequences used in this work were summarized in Table S1.

Table S1. The DNA sequences.

Probe ssDNA	5'-NH_2-$(CH_2)_6$-AGA GGT TGC CGT AGA GGT TGC CGT-3'
Target ssDNA	5'-NH_2-$(CH_2)_6$-ACG GCA ACC TCT ACG GCA ACC TCT-3'
ncDNA1	5'-NH_2-$(CH_2)_6$-AGT TCG CTG GTC AGA GCT TTC GGA-3'
ncDNA2	5'-NH_2-$(CH_2)_6$-AGA GCT TTC GGA AGT CTA TCG GTT-3'

2. The Fourier-Transform Infrared Spectrum (FTIR) of UCNPs

The Fourier-transform infrared spectrum (FTIR) of UCNPs (Figure S1) shows four obvious peaks around 1090, 1400, 1617, and 3420 cm^{-1}, representing the stretching vibrations of C-O, C-H, C=O, and O-H bonds, respectively. UCNPs are inorganic nanomaterials that do not demonstrate distinctive peaks in the FTIR spectrum. On the other hand, polyethylene glycol (PEG) contains functional groups of C-O, C-H, and O-H, but lacks a C=O functional group. The presence of peaks corresponding to C=O and O-H functional groups in the FTIR spectrum indicates that the surface of UCNP-PEG has been modified with carboxyl groups, which facilitates bonding with the target oligo.

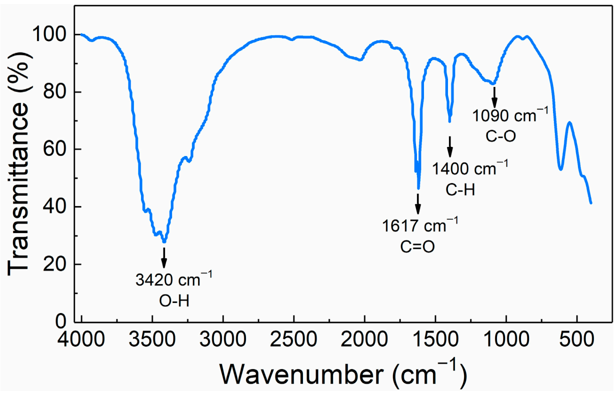

Figure S1. The Fourier-transform infrared (FTIR) spectrum of UCNPs coated with PEG-COOH.

3. The Photoluminescence (PL) Spectrum and UV-vis Absorption Spectrum of UCNP-labeled Target DNA

UCNPs exhibit fluorescence at around 450 nm and 475 nm under the excitation of 980 nm NIR light. DNA has a characteristic absorption peak at 260 nm. After UCNPs are connected with target DNA, the UCNP-labeled target DNA shows both PL peaks at around 450 nm and 475 nm (Figure S2a) and absorption peak at 260 nm (Figure S2b).

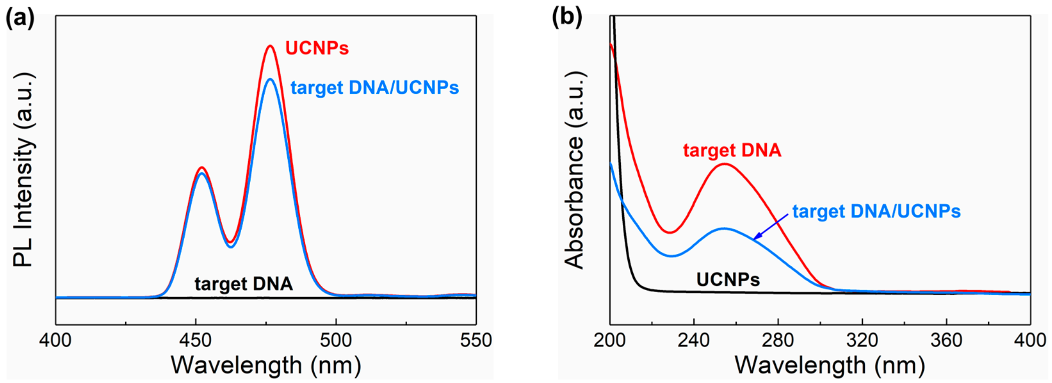

Figure S2. The photoluminescence (PL) spectrum (a) and UV-vis absorption spectrum (b) of UCNP-labeled target DNA.

4. The Stability of the UCNP-Powered PEC Bioanalysis

Six sets of assays were conducted under identical conditions, with a target DNA concentration of 1 μM. The photocurrents of the electrodes showed minimal variation at each step involving the modification of CdS QDs, ssDNA, and dsDNA. These results serve to validate the reproducibility and stability of the detection platform.

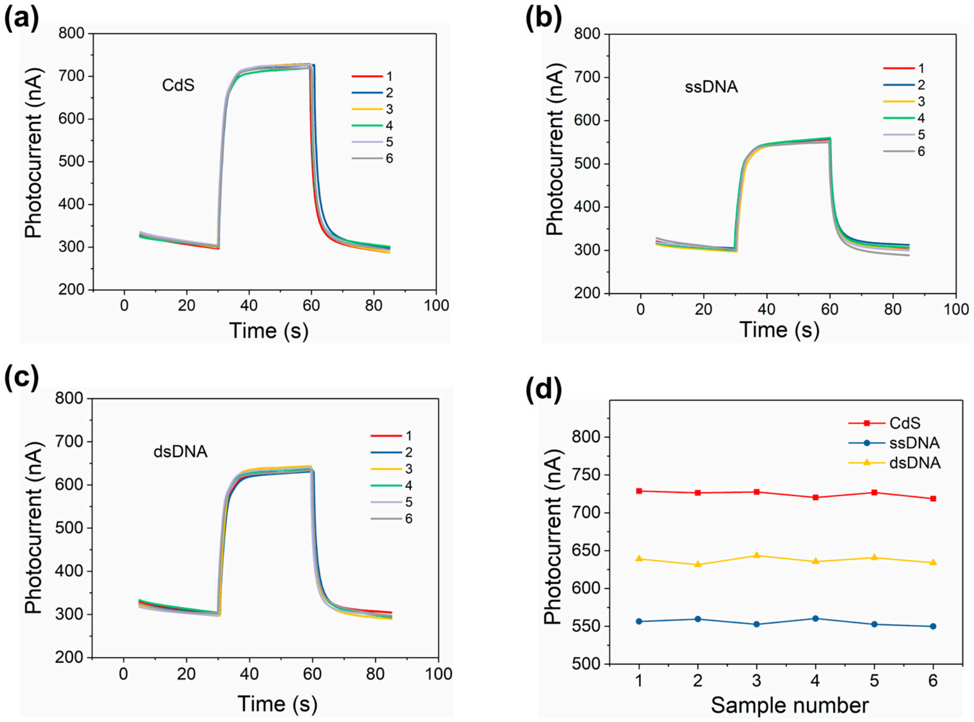

Figure S3. The photocurrent of six electrodes with the modification of (a) CdS QDs, (b) ssDNA, and (c) dsDNA (1 μM). (d) Comparison of the photocurrent of six work electrodes at each step.
